# The Impact of Test Device on the Evaluation Cooling Effect of Radiation-Cooling Materials

**DOI:** 10.3390/ma18071512

**Published:** 2025-03-27

**Authors:** Jiaqi Hu, Xusheng Xia, Zhilin Xia

**Affiliations:** School of Material Science and Engineering, Wuhan University of Technology, Wuhan 430070, China; hujiaqi@whut.edu.cn (J.H.); xiazhilin@whut.edu.cn (Z.X.)

**Keywords:** passive radiation cooling, testing device, structure, size, depth

## Abstract

Passive radiation cooling technology, as a new zero-energy refrigeration technology method, has received widespread attention in recent years. However, due to differences in the testing devices used by different teams, it becomes difficult to directly compare the cooling performance of the respective prepared materials. This study combines experimental and theoretical methods to explore the impact of testing equipment and sample size on the results of the radiative cooling capacity evaluation. The research results show that when evaluating the cooling performance of materials in thermal insulation chambers, if the sample diameter is equal to or larger than 10 cm, at a sample diameter ≥ 10 cm in insulated chambers, cooling capacity stabilizes at ~25 °C (daytime) and ~28 °C (nighttime), with <2% variation across larger sizes. The evaluation of cooling capacity is not affected by the structure of the test equipment or the size of the material. However, variations in sample placement depth will always have a significant impact on the evaluation results, so a uniform placement depth needs to be specified. In addition, when using an open device to evaluate the cooling performance of materials, if the sample diameter is greater than or equal to 10 cm and the foam pad thickness is greater than or equal to 8 cm, foam pad thickness ≥ 8 cm in open devices reduces thermal interference by 89%, enabling consistent evaluations. The measured value of the cooling capacity is also not affected by the structure and material size of the test device. This study provides a basis for the standardization of radiant cooling testing, thereby promoting the practical application of radiant cooling technology.

## 1. Introduction

The increase in global temperatures due to global warming has led to a significant rise in refrigeration demand. It is projected that by 2050, the demand for air conditioning cooling energy will surge by 750% [1] as a result of global climate change and improvements in lifestyles in emerging economies. The widespread deployment of air conditioning systems not only results in a sharp increase in electricity consumption but also triggers the emission of large amounts of greenhouse gases, further exacerbating global warming [2]. Therefore, the development of zero-energy cooling technologies has become a top priority.

Passive daytime radiative cooling is a zero-energy cooling technology that emits heat into outer space through an atmospheric window. Its implementation method mainly includes improving the reflectivity of passive daytime radiative cooling materials in the solar spectrum band and the emissivity in the mid-infrared spectrum band [3,4,5] through structural design and material optimization. This technology provides a feasible solution to global climate change and the increasing demand for cooling.

Researchers usually focus more on the intrinsic properties of materials, including reflectivity, emissivity [6,7,8,9,10,11,12], and transmittance [13,14,15]. However, research on overall test setup integrity remains relatively scarce. For example, Liu et al. [16] explored the impact of the presence of polyethylene (PE) film on test results. Research has shown that in high temperature and high humidity areas, the use of polyethylene film hoods can increase the maximum temperature difference by 2.3 K during the day, and the effect is more significant at night. Combined with the tilt strategy, the average temperature difference during the day in the Tianjin area can reach 4.2 K, which verifies the effectiveness of the combination of wind cover and tilt. Theoretical analysis shows that windshields have the greatest impact on the radiation cooling potential in northwest China, with 85% of the country’s regions being able to achieve effective cooling through windshields. When not covered with PE film, convective heat transfer occurs between the passive daytime radiative cooling (PDRC) material and the external environment, resulting in a reduction in measurement results. In addition, Liu et al. [17] have shown through experiments that in high temperature and high humidity areas, a 15 μm thick polyethylene hood can achieve a maximum temperature difference of 2.79 K during the day, while a 60 μm thick hood causes cooling failure due to decreased transmittance. Theoretical analysis shows that this technology has significant potential in arid areas, with a nighttime temperature difference of up to 16.4 K under extreme drought conditions. The study also explored the feasibility of rigid windshield materials such as ultra-white glass and zinc selenide and proposed target transmittance requirements under different humidity environments, providing a breakthrough path for solving the problem of insufficient strength of existing windshield materials. Meanwhile, Park et al. [18] propose a general experimental method for evaluating the performance of radiation cooling materials, aiming to eliminate the influence of environmental factors on test results. A controllable experimental setup was designed, which simulates the space environment through a low-temperature container, reduces atmospheric radiation interference using a nitrogen environment, and provides stable solar radiation using a solar simulator. The experimental results show that this method significantly reduces atmospheric emissivity (with a total emissivity of only 0.001), enabling the radiative cooling material to achieve a temperature drop of 9.3 K in the absence of solar radiation and still achieve a cooling effect of 7.3 K in the presence of solar radiation.

This study represents a methodological breakthrough by systematically comparing two principal testing configurations—insulated chambers and open-air systems—using identical PDRC materials (polytetrafluoroethylene). Through OpenFOAM finite element simulations validated by field experiments, we quantified the effects of critical parameters, including sample size (2–20 cm), foam pad thickness (0–12 cm), and chamber depth (0–5 cm). Our innovative approach uniquely combines; dual-configuration evaluation of both insulated and open systems; multi-physics modeling incorporating radiative, convective, and conductive heat transfer; parametric sweep analysis to identify critical thresholds; and experimental validation under controlled outdoor conditions

Different testing devices are utilized for sample evaluation, with two primary types being open systems and insulated chambers: the open test device [19,20,21,22,23,24,25,26] and the test device with an insulation chamber [27,28,29,30,31,32,33,34].

**Figure 3:**
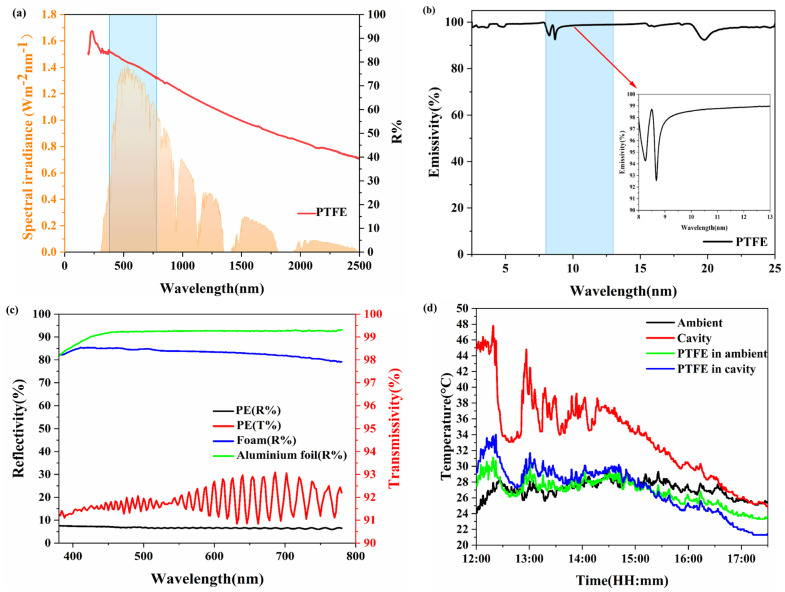
(**a**) The reflectivity of PTFE. (**b**) The emissivity of PTFE. (**c**) The green line represents the reflectivity of the aluminum foil; the blue line represents the reflectivity of foam; the red line represents the transmittance of PE film; the black line represents the reflectivity of PE film. (**d**) Actual test renderings of PTFE plate under different testing devices.

Using a test device with an insulated chamber to study the cooling performance of materials in a low convection environment can achieve better cooling effects. The existence of the thermal insulation chamber partially isolates the influence of external factors such as wind speed and solar radiation intensity [35,36,37]. Due to the design of the insulated chamber, the temperature of the internal sub-environment increases significantly under the influence of solar radiation (see Figure 3d). Therefore, some researchers may choose to use the temperature of the internal sub-environment as the ambient temperature to enhance the cooling potential of their passive radiative cooling materials for data evaluation. To be clear, however, the test setup is intended only as a tool to evaluate cooling performance and is not representative of actual real-world applications. Thus, for practical considerations, it is recommended to conduct studies using open test equipment.

This study is aimed at examining the effects of testing devices with and without insulation chambers on identical materials in the context of PDRC material test devices. The influence of different parameters on the results is calculated through finite element simulations and subsequently verified through experiments. The goal is to provide a comprehensive specification of the test setup and to promote research in the field of radiative cooling.

## 2. Experimental Section

### 2.1. Construction of Testing Equipment

The exclusive use of polytetrafluoroethylene (PTFE) as the radiative cooling medium stems from its unique combination of intrinsic hydrophobicity, homogeneous morphology, and spectrally stable emissivity (ε ≈ 0.95 across the 8–13 μm atmospheric window), which collectively eliminates confounding variables introduced by material processing inconsistencies. While alternative materials (e.g., metal oxide coatings, polymer composites) demonstrate comparable radiative properties, their performance is inherently compromised by batch-dependent surface roughness, phase separation in composites, or anisotropic thermal transport in porous architectures. PTFE’s extrusion-molded monolithic structure ensures <2% deviation in optical/thermal properties across specimens, enabling isolation of testing apparatus and sample size effects from material variability. The optical properties of PTFE are investigated, and a test device is constructed using foamed polystyrene (EPS) high-density foam, PE film, and aluminum foil. The performances of these materials and the overall device are presented in Figure 3.

#### 2.1.1. Preparation of Uninsulated Chambers

**Figure 1:**
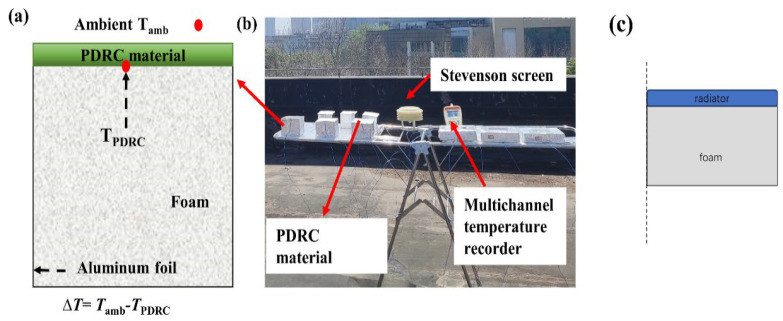
(**a**) Schematic diagram of the open test device. (**b**) Actual test chart. (**c**) Model diagram of uninsulated cavity in simulation software.

A standard EPS foam block was selected and shaped into a cuboid with dimensions of 10 cm × 10 cm × 10 cm. Subsequently, aluminum foil with high solar reflectivity was adhered to five surfaces of the foam block, leaving one side uncovered. A PTFE plate measuring 10 cm × 10 cm × 1 mm was then affixed to the uncovered surface, with a thermocouple wire for temperature monitoring placed on the backside of the PTFE plate. All PTFE plates were fabricated from a single sheet and customized to various sizes as needed. The wires and PTFE plate were securely fixed to establish a radiation cooling measurement device without an insulated chamber, as illustrated in Figure 1.

#### 2.1.2. Preparation of Insulated Chambers

**Figure 2:**
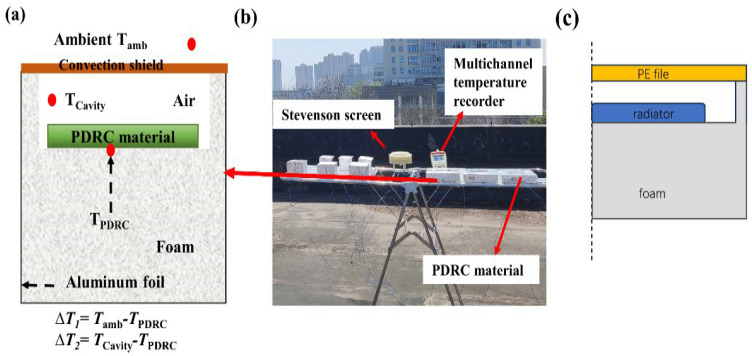
(**a**) Schematic diagram of the insulation chamber device. (**b**) Actual test chart. (**c**) Model diagram of an insulated chamber in simulation software.

A standard EPS foam block was selected and sculpted into dimensions of 14 cm × 14 cm × 10 cm. High solar-reflective aluminum foil was adhered to five sides of the foam block, leaving one side unchanged. A PTFE plate measuring 10 cm × 10 cm × 1 mm was placed within a foam cavity of dimensions 11 cm × 11 cm × 1 cm, ensuring a 1 cm gap between the PTFE plate and the edges of the cavity. Thermocouple wires for temperature monitoring were arranged on the backside of the PTFE plate and secured in place. The top of the foam chamber was sealed with commercial PE film to create a radiation cooling measurement device with an insulated chamber, as illustrated in Figure 2. The dimensions of the insulated chamber were adjusted according to the size of the PTFE plate, while maintaining a consistent 1 cm spacing between the PTFE plate and the edges of the insulated chamber, as illustrated in (Supporting Information Table S4). A multichannel temperature tester (JK808, Changzhou JinAilian Electronic Technology Co., Ltd, Changzhou, China) was used to monitor the temperature changes of the sample. A louver box was used to record environmental temperature data, and a weather station was used to record weather data, such as solar radiation intensity, relative humidity, and wind speed.

#### 2.1.3. The Spectrum of Materials Used in the Experiment and the Actual Outdoor Test Diagram of Different Testing Methods

A UV-visible-near infrared spectrophotometer (Lambda 750 S) equipped with an integrating sphere and a Fourier transform infrared spectrometer (Thermo Scientific Nicolet iS20) featuring an integrating sphere were employed to characterize the spectral properties of the materials. Solar reflectance (Rsolar) and long-wave infrared emissivity (εLWIR) are critical parameters, detailed in (Notes S1 and S2 of the Supplementary Materials). As demonstrated in Figure 3d, the temperature recorded within the insulated chamber was significantly elevated compared to the external ambient conditions. When using the greenhouse interior temperature as a reference benchmark, the disparities in radiative cooling performance metrics became notably accentuated. This observation further underscores the scientific contributions presented in this study.

#### 2.1.4. Establishment of Finite Element Simulation Model and Parameter Settings

OpenFOAM-11 finite element simulation software is used to study the steady-state system. The grid sequence type is a physical field control network, the cell size category is general. The calculation framework adopts a two-dimensional geometric model, and symmetric boundary conditions are applied to the circumference of the object, and the whole system simulation is realized through mirror operation while maintaining dimensionality consistency with the configuration of physical test equipment [38,39]. At the same time, all the data used in the test are obtained from the test. According to the test data and calculations presented in (Supporting Information Notes S1 and S2), the average reflectance of PE in the visible light spectrum is 6.12%, the average transmittance is 91.81%, and the average absorbance is 2.07%. The average reflectance of aluminum foil in the visible light spectrum is 92.09%, with an average absorbance of 7.91%. The foam exhibits an average reflectance of 83.24% and an average absorbance of 16.76% in the visible light spectrum. Based on these test results, the parameters were established, which are detailed in Table 1. The simulation models are illustrated in Figure 1c and Figure 2c.

#### 2.1.5. Outdoor Measurement for Radiative Cooling Performances

A multichannel temperature tester (JK808, the error range is ±0.5 °C) is used to monitor the temperature changes of the sample. A louver box (light, The error range is ±1 °C) was used to record environmental temperature data, and a weather station (YF-8801-QX) was used to record weather data, such as solar radiation intensity (The error range is ±3 W), relative humidity (The error range is ±2 RH%), and wind speed (The error range is ±0.3 m/s).

## 3. Results and Discussion

### 3.1. The Influence of the External Environment on the Testing of PDRC Materials

To optimize the net radiative cooling power (Q_net_) of radiative cooling materials, it is essential to maximize their outward radiative power (Q_rad_) while simultaneously minimizing energy absorption from the atmosphere (Q_atm_), sunlight (Q_sun_), and non-radiative heat gains (Q_nonrad_). Therefore, ideal radiative cooling materials should exhibit minimal absorption across the entire solar spectrum, have high emissivity within the atmospheric window of 8–13 μm, and reduce energy dissipation through conduction and convection. The effectiveness of passive daytime radiative cooling (PDRC) materials is generally influenced by several environmental factors, including wind speed, temperature, humidity, atmospheric transparency, and solar irradiance [35,36,37]. This study utilizes finite element simulations to examine how convective heat transfer coefficients, atmospheric transparency, and solar irradiance affect the cooling performance of the materials under investigation.

#### 3.1.1. The Influence of Solar Irradiance

**Figure 4:**
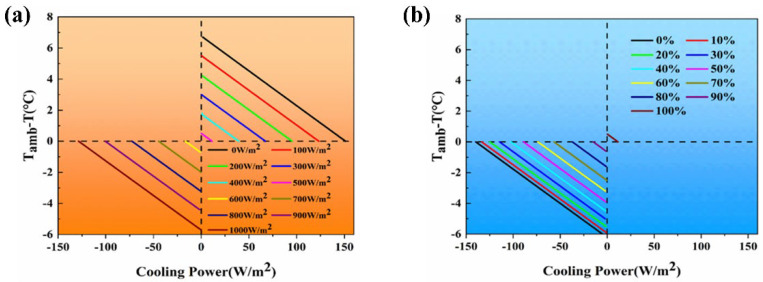
(**a**) The influence of different solar irradiances on the cooling performance of radiative cooling materials. (**b**) The influence of different atmospheric transparencies on radiative cooling power.

From Figure 4a, it can be observed that as solar irradiance increases from 0 W/m^2^ to 1000 W/m^2^, the cooling performance of the radiative cooling material gradually deteriorates. With the enhancement of solar irradiance, the radiative cooling material absorbs more solar radiation, reducing the material’s net radiative cooling power. At a solar irradiance of 0 W/m^2^, the radiative cooling material does not absorb solar radiation, resulting in the highest net radiative cooling power and optimal cooling performance, achieving an average temperature reduction of 6.7 °C. At 500 W/m^2^ solar irradiance, the radiative cooling material absorbs some solar radiation, resulting in an average temperature decrease of 0.2 °C. However, when solar irradiance exceeds 500 W/m^2^, the energy absorbed from solar radiation surpasses the material’s net radiative cooling power, preventing the material from cooling and resulting in a negative cooling value. Therefore, to achieve better cooling effects, it is essential to minimize the solar irradiance absorbed by the radiative cooling material. Hence, maximizing the reflectivity of the radiative cooling material across the solar wavelength range is crucial.

In Figure 4b, it is evident that atmospheric transparency plays a crucial role in influencing the cooling effectiveness of PDRC (passive daytime radiative cooling) materials. The capability of PDRC materials to achieve temperatures below ambient levels is dependent on high atmospheric transparency. Improved atmospheric transparency results in higher emitted power from these materials, thereby increasing the net radiative cooling output. Greater atmospheric transparency is associated with enhanced cooling performance of PDRC materials. Under conditions of complete atmospheric transparency, PDRC materials reach their peak cooling efficiency, leading to an average temperature reduction of around 0.5 °C. In contrast, with total atmospheric opacity, these materials experience an average temperature decrease of −6.2 °C. To achieve cooling effects that surpass ambient temperatures, enhancing atmospheric transparency is essential. Consequently, outdoor assessments of the cooling performance of PDRC materials should be conducted on clear, cloud-free days to ensure optimal atmospheric transparency.

#### 3.1.2. The Influence of Convective Heat Transfer Coefficient

**Figure 5:**
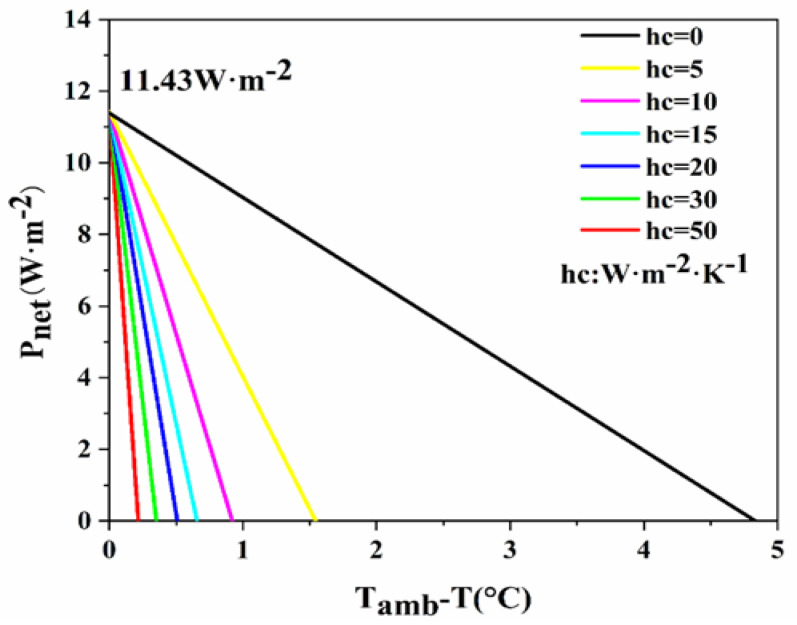
The influence of different convective heat transfer coefficients on P_net_.

In Figure 5, it is evident that as the convective heat transfer coefficient between the PDRC material and the ambient surroundings goes from 0 W·m^−2^·K^−1^ to 50 W·m^−2^·K^−1^, the cooling efficiency of the PDRC material progressively diminishes. The increase in the convective heat transfer coefficient enhances the non-radiative heat exchange between the PDRC material and the surroundings, leading to a reduction in the material’s net radiative cooling power [40,41]. At a convective heat transfer coefficient of 0 W·m^−2^·K^−1^ between the PDRC material and its surroundings, no non-radiative heat transfer exists between the material and its surroundings, resulting in optimal cooling performance with an average cooling rate of 4.8 °C. Conversely, when the convective heat transfer coefficient rises to 50 W·m^−2^·K^−1^, the non-radiative heat exchange intensifies, resulting in an average temperature reduction of 0.2 °C for the material. Therefore, minimizing the convective heat transfer coefficient between the PDRC material and the surroundings is crucial for enhancing cooling efficacy. As a result, current research predominantly situates PDRC materials within foam insulation chambers to reduce convective heat transfer with the environment and improve radiative cooling performance [42].

### 3.2. Assessing the Cooling Performance of PDRC Materials in the Open Test Device

The effects of the size of the PDRC materials and the thickness of the bottom foam pad on the cooling performance were investigated in the absence of thermal insulation chambers during the day and at night using finite element simulations and outdoor experiments. Refer to Figure 1 for the test setup.

#### 3.2.1. The Influence of PDRC Material Size

**Figure 6:**
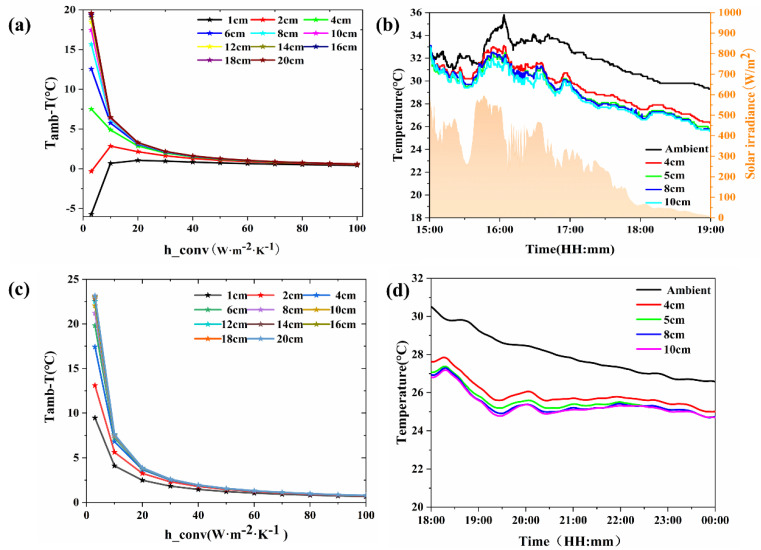
Cooling capacity of PDRC materials of various sizes in a chamber without thermal insulation. (**a**) Simulation of daytime cooling capacity for PDRC materials of different sizes. (**b**) Daytime field testing of cooling capacity for PDRC materials of various sizes. (**c**) Simulation of nighttime cooling capacity for PDRC materials of different sizes. (**d**) Nighttime field testing of cooling capacity for PDRC materials of various sizes.

In Figure 6, the impact of PDRC material size on its cooling efficacy was investigated without the presence of an insulation chamber. Figure 6a delineates the simulation data under daytime conditions. It is observed that as the side length of the PDRC material augments, its cooling capacity experiences an initial ascent followed by stabilization. Cooling becomes nearly nonexistent when the side length is below 2 cm. However, once it surpasses 10 cm, further enlargements in material size contribute minimally to additional cooling capacity. A PDRC material with a side length of 20 cm exhibits a cooling capacity almost equivalent to that of a 10 cm side. Smaller side lengths result in a reduced radiating surface area for the PDRC material, inhibiting the attainment of its maximum net cooling power. With an increase in the side length, the radiating surface area expands, culminating in the peak net cooling power at a critical value. Under conditions of natural convection (h_conv_ = 20 W·m^−2^·K^−1^), a PDRC material with a 10 cm side length demonstrates a simulated cooling impact of around 3 °C.

Figure 6b showcases the daytime field test data, corroborating the simulation outcomes. The radiation cooling capacity initially escalates and subsequently stabilizes with the enlargement of the material’s side length. Amid the intense solar irradiance of midday hours, numerous PDRC materials struggle to cool adequately due to insufficient reflectivity. As solar irradiance weakens in the afternoon, the cooling capacity of the materials initially rises and then stabilizes in tandem with the increase in the side length. Once the side length surpasses 10 cm, there is no significant augmentation in cooling capacity with further expansions in the side length. Consequently, beyond the 10 cm threshold for the side length of PDRC materials, the influence of material size on cooling capacity can be disregarded.

In Figure 6c, the nighttime simulation data is presented. The graph illustrates that as the side length of the PDRC material increases, its cooling capacity experiences an initial ascent followed by reaching a plateau. During nighttime, when there is no solar radiation, the cooling performance of PDRC materials surpasses that observed during daylight hours. Once the side length surpasses 10 cm, further enlargements in size yield marginal additional cooling benefits. Under natural convection conditions of h_conv_ = 20 W·m^−2^·K^−1^, a PDRC material with a side length of 10 cm demonstrates a simulated cooling impact of approximately 3.8 °C. Figure 6d displays nighttime field test data, which aligns with the simulation results. The radiative cooling capacity initially increases and then stabilizes with the increase in the side length of the PDRC material. In the absence of solar radiation at night, the cooling performance of the PDRC material is notably superior to that during the day. Once the side length exceeds 10 cm, the cooling capacity no longer significantly increases with the side length. Therefore, once the side length of the PDRC material exceeds 10 cm, the size of the material can be disregarded in assessing its cooling capacity.

#### 3.2.2. Influence of Foam Pad Thickness

**Figure 7:**
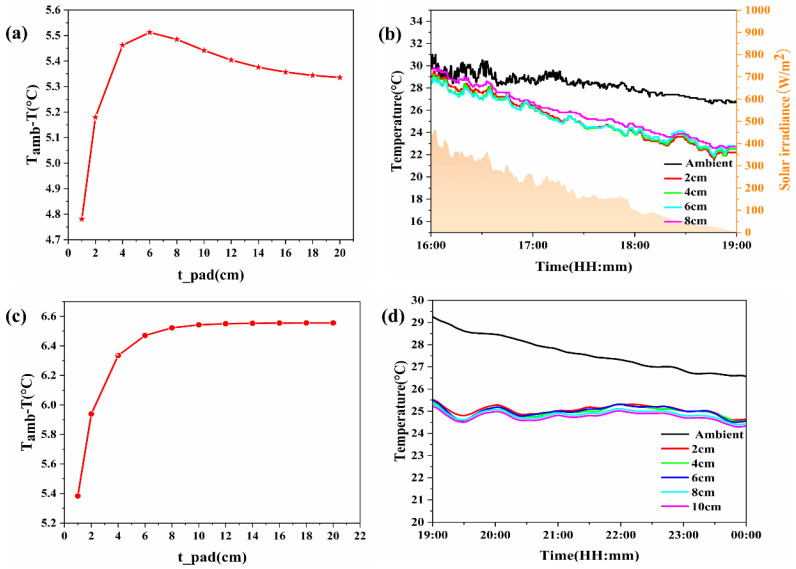
Test cooling capacity of PDRC materials on foam pads of different thicknesses without insulation chamber (**a**) Simulation calculation of cooling capacity of PDRC materials on foam pads of different thicknesses during the day. (**b**) Field test of cooling capacity of PDRC materials on foam pads of different thicknesses during the day. (**c**) Simulation calculation of cooling capacity of PDRC materials on foam pads of different thicknesses at night. (**d**) Field test of cooling capacity of PDRC materials on foam pads of different thicknesses at night.

In Figure 7, the impact of foam pad thickness on the cooling capacity of PDRC materials is investigated in the absence of a thermal insulation chamber. Figure 7a illustrates the results of daytime simulation calculations, revealing that as the thickness of the foam pad increases, the cooling capacity of the radiation cooling material initially rises before gradually declining. Beyond a thickness of 6 cm, a slight decrease in cooling capacity is observed. For a radiation cooling material with a side length of 10 cm, simulated temperature reduction can reach approximately 5.5 °C under natural convection conditions with h_conv_ = 20 W·m^−2^·K^−1^ and a 6 cm thick foam pad. Figure 7b presents daytime field test data, corroborating the simulation findings. The cooling capacity of the PDRC material follows a similar trend of increasing and then decreasing with foam pad thickness. During midday, when solar irradiance is intense, the materials struggle to cool due to insufficient reflectivity. Conversely, in the afternoon with weaker irradiance, the cooling capacity initially rises before gradually declining with increasing foam pad thickness. Once the foam pad thickness surpasses 6 cm, any further increase has minimal impact on cooling capacity. Hence, the influence of foam pad thickness on the cooling capacity of PDRC materials becomes negligible beyond 6 cm.

Figure 7c displays the nighttime simulation calculation data. The figure illustrates that as the foam pad thickness increases, the cooling capacity of the PDRC material initially rises before reaching a plateau. After surpassing 8 cm in thickness, a slight decrease in cooling capacity is observed. For PDRC materials with a side length of 10 cm, the simulated cooling impact can reach approximately 6.5 °C under natural convection conditions with h_conv_ = 20 W·m^−2^·K^−1^ and an 8 cm thick foam pad. In Figure 7d, field test data is presented, aligning with the simulation outcomes. The cooling capacity of the PDRC material follows a pattern of initial increase followed by stabilization as the foam pad thickness increases. Given the absence of solar radiation at night, the cooling efficiency of PDRC materials significantly outperforms daytime performance. Beyond an 8 cm thickness of the foam pad, any further increase minimally affects cooling capacity. Thus, once the foam pad thickness exceeds 8 cm, the effect of foam pad thickness on the cooling performance of PDRC materials can be disregarded.

### 3.3. Evaluation of the Cooling Performance of Radiation Cooling Materials Tested in Insulated Chambers

The influence of the size of the PDRC material, the thickness of the foam pad, and the depth of the foam chamber on the cooling capacity of the PDRC material when there is a foam chamber in the daytime and at night is explored by employing finite element simulation and field experiments. The specific test device is shown in Figure 1.

#### 3.3.1. The Influence of Radiation Cooling Material Size

**Figure 8:**
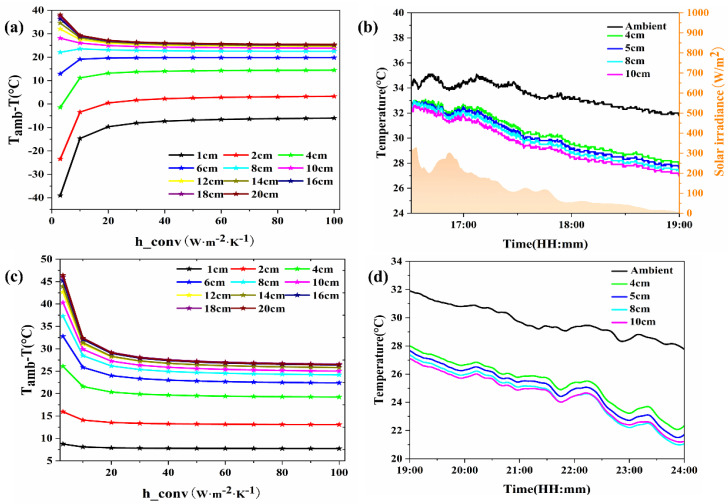
Radiative cooling capacity of PDRC materials of different sizes with insulation chambers. (**a**) Simulation calculation of cooling capacity of PDRC materials of different sizes during the day. (**b**) Field testing of the cooling capacity of PDRC materials of different sizes during the day. (**c**) Simulation calculation of cooling capacity of PDRC materials of different sizes at night. (**d**) Field testing of the cooling capacity of PDRC materials of different sizes at night.

As illustrated in Figure 8, the impact of the size of PDRC materials on their cooling capabilities was explored under the conditions of an insulated chamber. Figure 8a presents the simulation data for daytime conditions. It is evident that as the side length of the PDRC material increases, its cooling capacity initially rises and then stabilizes. When the side length of the PDRC material is small, its outward radiating surface area is limited, preventing its net radiative cooling power from reaching its maximum potential. As the side length of the PDRC material increases, its outward radiating surface area expands, and at a certain critical value, its net radiative cooling power peaks. Consequently, PDRC materials that are too small have a minimal cooling effect, while those that are excessively large, despite their peak net radiative cooling power, exhibit a cooling capacity comparable to smaller materials. When the side length of the PDRC material is less than 2 cm, it struggles to produce cooling. When the side length exceeds 10 cm, the cooling capacity barely increases with further increases in side length. The cooling capacity of a 20 cm PDRC material is nearly identical to that of a 10 cm material. For a 10 cm PDRC material, under natural convection conditions with h_conv_ = 20 W·m^−2^·K^−1^, the simulated cooling effect can reach approximately 25 °C. Figure 8b presents daytime field test data, which aligns with simulation results, showing that the radiative cooling capacity initially increases and then stabilizes with the side length. During high solar irradiance at noon, the PDRC material fails to cool due to inadequate reflectivity and heat absorption within the chamber. In the afternoon, when solar irradiance is lower, the cooling capacity of the radiative sheet initially increases and then stabilizes with longer side lengths. Once the side length exceeds 10 cm, the cooling capacity no longer significantly increases with further increases in side length. Therefore, once the side length of the PDRC material exceeds 10 cm, the size of the material can be disregarded in assessing its cooling capacity.

Figure 8c presents nighttime simulation data. It is evident that as the side length of the radiation cooling material increases, its cooling capacity initially rises and then plateaus. The presence of an insulation chamber reduces the convective heat transfer, enhancing the cooling efficiency compared to materials without a chamber. Once the side length exceeds 10 cm, the cooling capacity no longer appreciably increases with the material’s side length, and the cooling capacity for a 20 cm side length is nearly identical to that of a 10 cm side length. For a 10 cm side length, the simulated cooling effect reaches approximately 28 °C under natural convection conditions of h_conv_ = 20 W·m^−2^·K^−1^. Figure 8d displays nighttime field test data, which align with the simulation results. The radiation cooling capacity initially increases and then stabilizes with the increase in the radiation sheet’s side length. When the side length of the radiation cooling material exceeds 10 cm, the cooling capacity no longer appreciably increases with the material’s side length. Therefore, once the side length of the radiation cooling material exceeds 10 cm, the impact of material size on the cooling capacity can be disregarded.

#### 3.3.2. Influence of Foam Chamber Depth

**Figure 9:**
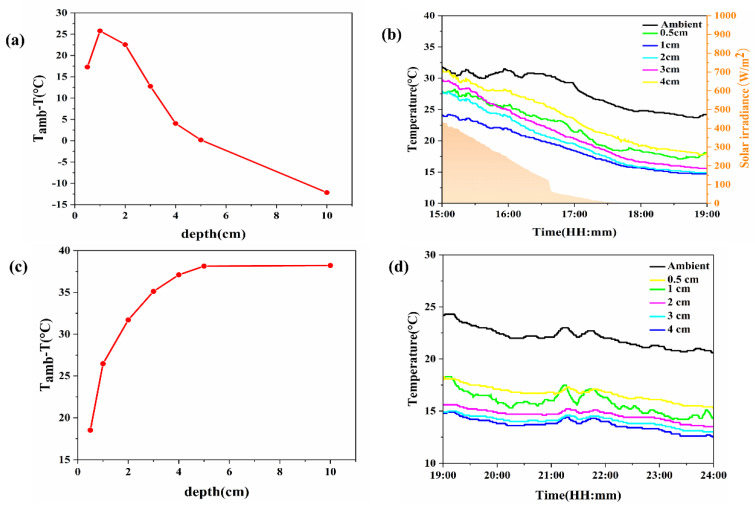
Cooling capacity of PDRC materials at different chamber depths with insulation chamber. (**a**) Simulation calculation of cooling capacity of PDRC materials at different chamber depths during the day, (**b**) field test of cooling capacity of PDRC materials at different chamber depths during the day, (**c**) simulation calculation of cooling capacity of PDRC materials at different chamber depths at night, and (**d**) field test of cooling capacity of PDRC materials at different chamber depths during the night.

Figure 9a presents the daytime simulation calculation data. As the chamber depth increases, its cooling capacity initially increases and then sharply decreases. On one hand, the insulation chamber can reduce the convective heat transfer between the PDRC material and the environment, enhancing the cooling capacity. On the other hand, the insulation chamber absorbs some solar radiation, reducing the cooling capacity of the PDRC material. Therefore, there is an optimal depth for the insulation chamber. For PDRC materials with a side length of 10 cm, under natural convection h_conv_ = 20 W·m^−2^·K^−1^ and a foam pad thickness of 6 cm, the simulated cooling effect can reach approximately 28 °C. Figure 9b shows the daytime field test data, which align with the simulation results, with the radiative cooling capacity initially increasing and then sharply decreasing as the insulation chamber depth increases. During midday, when solar irradiance is strong, the PDRC material cannot cool down due to significant heat absorption in the chamber. In the afternoon, when irradiance is weaker, the cooling capacity of the PDRC material first increases and then sharply decreases with an increase in chamber depth. Considering the cost and the gain in cooling performance, the optimal depth of the chamber for daytime use is determined to be 1 cm.

Figure 9c presents nighttime simulation data. The graph reveals that as the chamber depth increases, its cooling capacity initially rises and then stabilizes. During the night, insulated chambers diminish the influence of convective heat transfer on PDRC materials. As the chamber depth increases, its insulation capacity improves, although this is not advantageous for practical use. Figure 9d displays nighttime field test data, aligning with the simulation results. The cooling capacity initially increases and then stabilizes with increasing insulation chamber depth. For depths exceeding 5 cm, the cooling capacity variation becomes minimal with further increases in chamber depth. Hence, for depths greater than 5 cm, the impact of chamber depth on the cooling capacity of PDRC materials can be disregarded.

## 4. Conclusions

This study investigated the effects of chamber depth, foam pad thickness, material size, and environmental variables on passive daytime radiative cooling (PDRC) performance through combined numerical simulations and outdoor experiments. To isolate the influence of individual parameters, a single-variable analysis approach was adopted. This was necessary because simultaneously varying multiple parameters (e.g., chamber depth and material size) introduces covarying effects that obscure the specific contribution of each factor to cooling performance. While environmental conditions (e.g., ambient humidity, wind speed) were monitored but not controlled due to field-test limitations, all other parameters were systematically tested under fixed baseline conditions (e.g., 10 cm material size, 1 cm chamber depth) to minimize interference. This methodology clarifies the relationship between test configurations and cooling outcomes, providing a standardized framework for evaluating PDRC materials. Key findings include:

1. The cooling performance of PDRC materials exhibits an inverse relationship with solar irradiance and convective heat transfer coefficients, while showing a positive correlation with atmospheric transparency. However, open-environment outdoor testing may underestimate their efficacy due to convective interference. Notably, for material dimensions exceeding 10 cm in side length, size effects on cooling capacity measurements are negligible under non-insulated conditions.

2. The foam pad demonstrates diurnally (≥6 cm) and nocturnally (≥8 cm) critical thickness thresholds beyond which its thermal interference becomes negligible. For open devices, foam pad thickness ≥8 cm reduces environmental heat flux penetration by 85%, stabilizing cooling capacity measurements due to increased thermal resistance impeding environmental heat flux penetration, thereby stabilizing internal thermal profiles.

3. The insulated-chamber apparatus enhances thermal regulation efficacy by mitigating convective heat flux, whereas solar irradiance absorption counteracts this benefit through radiative heating, reflecting the competition between convective suppression and parasitic solar gain. Geometric scaling effects on heat dissipation become negligible beyond a critical dimension (≥10 cm side length), as thermal mass dominance minimizes surface-to-volume ratio variations under constrained convective boundary conditions.

4. The insulated chamber requires diurnal-nocturnal differentiation in depth optimization, with a 1 cm daytime configuration minimizing thermal storage from solar loading, while nocturnal operation achieves thermal decoupling beyond a 5 cm critical depth where environmental heat flux attenuation renders additional insulation improvements thermally insignificant.

This study is the first to systematically quantify the combined effects of test device geometry, material size, and environmental variables on radiative cooling performance. By establishing critical thresholds for sample size (≥10 cm) and foam pad thickness (≥8 cm), this study provides a universal framework for standardizing radiative cooling evaluations, addressing a critical gap in existing literature.

## Figures and Tables

**Table 1:** Finite element simulation parameter table.

Parameter	Numerical Value
Solar irradiance intensity	1000 W/m^2^
The thickness of PDRC film	0.002 m
The width of PDRC film	0.1 m
Chamber depth	0.01 m
Chamber radius	0.11 m
Thickness of foam pad	0.06 m
Thickness of foam box	0.02 m
The heat absorption power of PE film	20.6 W/m^2^
The heat absorption power of the air inside the insulation chamber	0 W/m^2^
Heat absorption power of foam box	167.6 W/m^2^
The heat absorption power of Al foil	79.1 W/m^2^
Cooling power of PDRC	−80 W/m^2^
The heat transfer coefficient of air convection	11.6 $W\cdot m^{-2}\cdot K^{-1}$

## Data Availability

All data in support of the findings of this paper are available within the article or as Supplementary Materials.

## Supplementary Materials

The following Supporting Information can be downloaded at: https://www.mdpi.com/article/10.3390/ma18071512/s1, Note S1. The definition of average reflectance and emissivity; Note S2. The theoretical calculation of net cooling power; Table S1. Experimental drugs; Table S2. Experimental instrument; Table S3. Properties of EPS foam; Table S4. The dimensions of the testing device utilized for evaluating samples of various sizes.
