# Supplementary material for: The Impact of Test Device on the Evaluation Cooling Effect of Radiation-Cooling Materials
Source: Materials (Basel). 2025 Mar 27;18(7):1512. doi: 10.3390/ma18071512 (PMC11989526; doi:10.3390/ma18071512)
Supplement: Supplementary file 1 [file materials-18-01512-s001.zip › materials-3537734-supplementary.pdf]

Supporting Information

The impact of test device on the evaluation cooling effect of radiation-cooling materials

Note 1. The definition of average reflectance and emissivity:

The average solar reflectance ($\bar{R}_{\text{solar}}$), denoted as:

$$\bar{R}_{solar} = \frac{\int_{0.3\mu m}^{2.5\mu m} I_{solar}(\lambda) R_{solar}(\lambda) d\lambda}{\int_{0.3\mu m}^{2.5\mu m} I_{solar}(\lambda) d\lambda} \tag{1}$$

In Eq. (1), λ represents the solar light band from 0.3-2.5 μm, $I_{\text{solar}}(\lambda)$ is the normalized ASTM G173 global solar intensity spectrum, and $R_{\text{solar}}(\lambda)$ is the spectral reflectance of the device.

The average emissivity in the longwave infrared atmospheric transmission window, $\bar{\varepsilon}_{LWIR}$ is defined as:

$$\bar{\varepsilon}_{LWIR} = \frac{\int_{8\mu m}^{13\mu m} I_{BB}(T,\lambda) \varepsilon_{LWIR}(\lambda) d\lambda}{\int_{8\mu m}^{13\mu m} I_{BB}(T,\lambda) d\lambda} \tag{2}$$

In Eq. (2), $I_{\text{BB}}(T,\lambda) = \frac{2hc^2}{\lambda^5} \frac{1}{e^{hc/\lambda k_B T} - 1}$, is the spectral radiation intensity of a black body at temperature T (assumed to be 25 °C) as defined by Planck's Law. h is Planck's constant, k_B is Boltzmann's constant, c is the speed of light in a vacuum, and $\varepsilon_{LWIR}(\lambda)$ is the emissivity of the device in the atmospheric window range of 8-13 μm.

The emissivity of the material, $\varepsilon(\lambda)$, is given by Kirchoff's law of thermal radiation:

$$\varepsilon(\lambda) = \alpha(\lambda) = 1 - \tau(\lambda) - R(\lambda) \tag{3}$$

In Eq. (3), $\alpha(\lambda)$ is the absorption rate, $\tau(\lambda)$ is the transmission rate, $R(\lambda)$ is the reflectance rate.

Note 2 The theoretical calculation of net cooling power:

$$P_{net}(T) = P_{rad}(T) - P_{sun} - P_{atm}(T_{atm}) - P_{cond+conv}(T, T_{atm}) \tag{4}$$

$$P_{\mathrm{rad}}(T) = A\int d\Omega \cos\theta \int_0^{\infty} d\lambda\, I_{BB}(T,\lambda)\varepsilon(\lambda,\theta) \tag{5}$$

$$P_{\mathrm{sun}} = \int_0^{\infty} I_{\mathrm{solar}}(\lambda)\big(1 - R_{\mathrm{solar}}(\lambda)\big)d\lambda \tag{6}$$

$$P_{\mathrm{atm}}(T_{atm}) = A\int d\Omega \cos\theta \int_0^{\infty} d\lambda\, I_{BB}(T_{atm},\lambda)\varepsilon(\lambda,\theta)\varepsilon_{atm}(\lambda,\theta) \tag{7}$$

$$P_{cond+conv} = h_c(T_{amb} - T) \tag{8}$$

In the context of a zero-energy switchable radiative cooler operating in cooling mode, $P_{net}(T)$ denotes the net cooling power of the device, P_{rad} is the power radiated outwards by the device, P_{sun} stands for the power absorbed from solar radiation by the device, P_{atm} represents the power absorbed from atmospheric radiation by the device, and $P_{cond+conv}$ signifies the power from non-radiative heat exchange processes such as conduction and convection. The terms T and T_{atm} correspond to the device temperature and the ambient temperature, respectively.

For Equation (4), under the condition that the temperature (T) of the zero-energy switchable radiative cooler reaches a steady state, the aforementioned parts achieve thermal equilibrium. In Equation (5), $\int d\Omega \cos\theta$ is the radiation angle integral over the hemispherical space.

In Equation (7), the term $\varepsilon_{atm}(\lambda,\theta) = 1 - \tau_{atm}(\lambda,\theta) = 1 - \tau(\lambda)^{1/\cos\theta}$ denotes the angle-dependent spectral emissivity of the atmosphere. Here, $\tau_{atm}(\lambda,\theta)=\tau(\lambda)^{1/\cos\theta}$ represents the angle-dependent atmospheric transmittance, and $\tau(\lambda)$ indicates the atmospheric transmittance in the zenith direction.

Regarding Equation (8), h_c is the non-radiative heat exchange coefficient resulting from the combined conduction and convection heat exchange between the device and the surrounding air. Its range can be restricted between 0 and 12 W/m^2/K.

Table S1 Experimental drugs

Drugs	Specifications	Manufacturer
PTFE plate	Thickness 1mm	Kean Sealing Materials Co., LTD
EPS Foam	high density	Dongguan Taolik building decoration materials Co., LTD
deionized water	0.5 us/cm	
PE film	Thickness 25 μm	Jiangmen Henghou plastic products Co., LTD
aluminum foil	Thickness 18 μm	Shanghai Yong Wang Packaging Products Co., LTD

Table S2 Experimental instrument

Name of the instrument	Product	Manufacturer
Temperature tester	JK-808	Changzhou Jinailian Electronic Technology Co., Ltd
Experimental pure water machine	AJF-0501-P	Yiyang Enterprise Development Co., Ltd
weather station	YF-8801-QX	Shenzhen Huayi Intelligent Measurement Technology Co., Ltd
Louver box	light	Shandong Renke Measurement and Control Technology Co., Ltd

Table S3 Properties of EPS foam

Attribute	Numerical value	Unit
Thermal conductivity coefficient	0.04	$W \cdot m^{-1} \cdot K^{-1}$
Constant pressure heat capacity	1500	$J \cdot kg^{-1} \cdot K^{-1}$
density	20	$kg \cdot m^{-3}$

Table S4 The dimensions of the testing device utilized for evaluating samples of various sizes.

Sample size（PTFE）	cavity of dimension (insulated chambers)	Open test
4cm× 4cm×1mm	6cm×6cm×10cm	4cm×4cm×10cmm
5cm×5cm×1mm	7cm×7cm×10cm	5cm×5cm×10cm
8cm×8cm×1mm	10cm×10cm×10cm	8cm×8cm×10cm
10cm×10cm×1mm	12cm×12cm×12cm	10cm×10cm×10cm
